# Bradycardic Tamponade: The Myxedema Pitfall

**DOI:** 10.7759/cureus.104017

**Published:** 2026-02-21

**Authors:** Mazen Ahmed, Ali Mohamedshata, Mohamed O Elhussain, Mohammed Elmojtaba Ahmed, Ali Ghandour

**Affiliations:** 1 Internal Medicine, Corewell Health Dearborn Hospital, Dearborn, USA; 2 Internal Medicine, Wayne State University Detroit Medical Center, Detroit, USA; 3 Cardiology, Corewell Health Dearborn Hospital, Dearborn, USA

**Keywords:** bradycardia, cardiac tamponade, echocardiography, hypothyroidism, myxedema, pericardial effusion, pericardiocentesis

## Abstract

Severe, untreated hypothyroidism can produce large, slowly accumulating pericardial effusions; progression to cardiac tamponade is uncommon and may present without tachycardia because of blunted sympathetic tone. We report a middle-aged woman with long-standing hypothyroidism who presented with dyspnea and hypotension but a low resting heart rate. Transthoracic echocardiography demonstrated a large circumferential effusion with right ventricular diastolic collapse and marked respiratory variation in tricuspid inflow, confirming tamponade physiology. She underwent urgent, echo-guided pericardiocentesis with the removal of a large volume of straw-colored fluid, followed by short-term catheter drainage and the initiation of thyroid hormone replacement. Cultures and cytology were negative, and the clinical course, with rapid hemodynamic recovery and resolution of the effusion, supported hypothyroidism as the most likely etiology after excluding infectious, malignant, and autoimmune causes. This case underscores a practical pitfall: bradycardia does not exclude tamponade in severe hypothyroidism. Diagnosis should be anchored in echocardiographic findings, and timely pericardial drainage coupled with endocrine management can achieve rapid stabilization and help prevent recurrence.

## Introduction

Hypothyroidism has diverse cardiovascular manifestations, including sinus bradycardia, impaired diastolic function, and pericardial effusion [1]. Pericardial effusion is a recognized consequence of long-standing disease and often accumulates slowly enough to remain clinically silent until pericardial reserve is exceeded [1,2]. The pathophysiology involves increased capillary permeability, interstitial glycosaminoglycan deposition, and reduced lymphatic drainage, which favor gradual fluid accumulation [1]. Although large effusions are not uncommon in this setting, progression to hemodynamic compromise is relatively rare; when tamponade occurs, compensatory tachycardia may be attenuated because sympathetic tone and β-adrenergic responsiveness are blunted, a "myxedema heart" phenomenon that can obscure recognition at the bedside [2]. Classically, tamponade presents with hypotension, elevated venous pressures, and reflex tachycardia (often with pulsus paradoxus), reflecting impaired diastolic filling; however, these bedside cues may be muted in severe hypothyroidism, making imaging pivotal [3].

Diagnosis relies on echocardiography demonstrating tamponade physiology [3,4], and management centers on urgent pericardial drainage with subsequent thyroid hormone replacement to address the underlying disorder and reduce the risk of recurrence [5-7]. This framework emphasizes that heart rate alone is an unreliable discriminator in myxedema-associated tamponade and that timely imaging-guided intervention with coordinated endocrine care is essential [8].

## Case presentation

**Figure 1:**
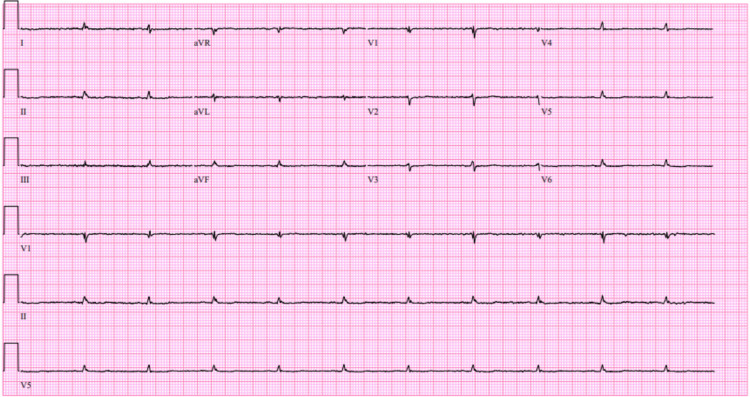
Twelve-lead electrocardiogram demonstrating normal sinus rhythm with low-voltage QRS complexes.

**Figure 2:**
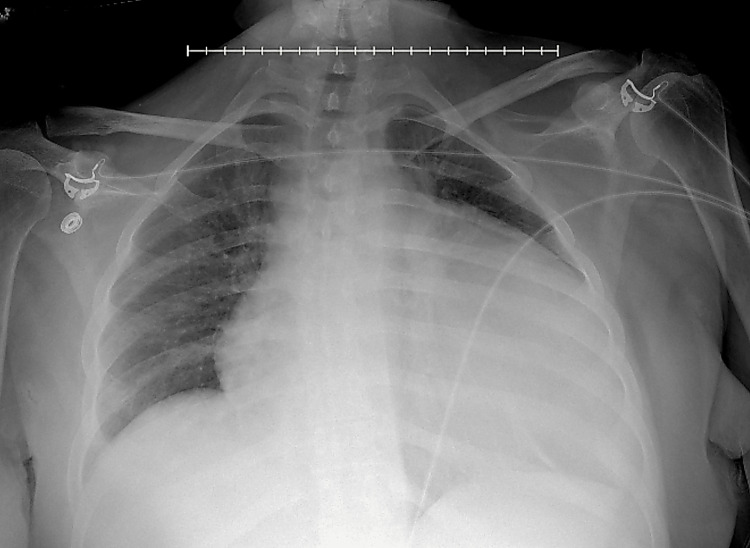
Portable AP chest radiograph demonstrating an enlarged cardiac silhouette. AP: anteroposterior

**Figure 3:**
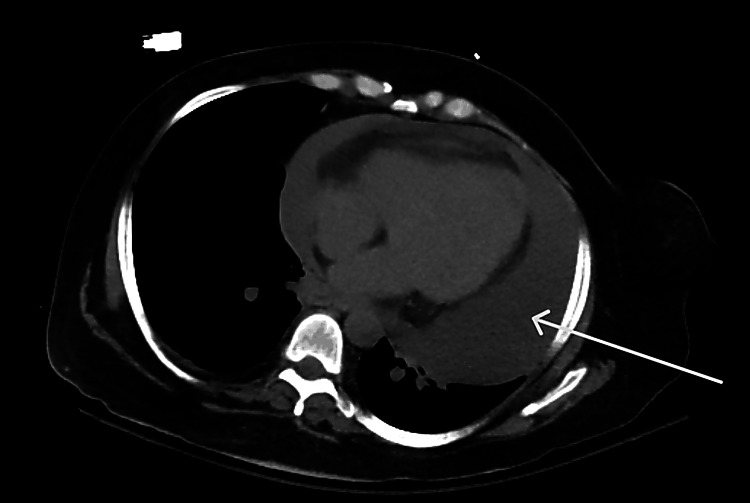
Axial CT scan (lower thoracic cut) showing a circumferential low attenuation surrounding the heart (arrow), compatible with a large pericardial effusion.

A 50-year-old woman with a long-standing history of hypothyroidism, who had not been taking her thyroid medication for years, presented with progressive dyspnea and fatigue. On arrival, blood pressure was 82/60 mmHg and heart rate 60 beats/min; examination was notable only for mild lower extremity edema. Pulsus paradoxus was not formally assessed. Laboratory testing confirmed severe hypothyroidism: thyroid-stimulating hormone (TSH) 65.04 μIU/mL (0.40-4.50) and free T4 <0.4 ng/dL (0.7-1.5). Triiodothyronine and thyroid peroxidase antibodies were not obtained. Electrocardiography showed a normal sinus rhythm with low-voltage QRS complexes (Figure 1). Chest radiography demonstrated an enlarged cardiac silhouette (Figure 2), and abdominal/pelvic CT incidentally identified a large pericardial effusion (Figure 3).

**Figure 4:**
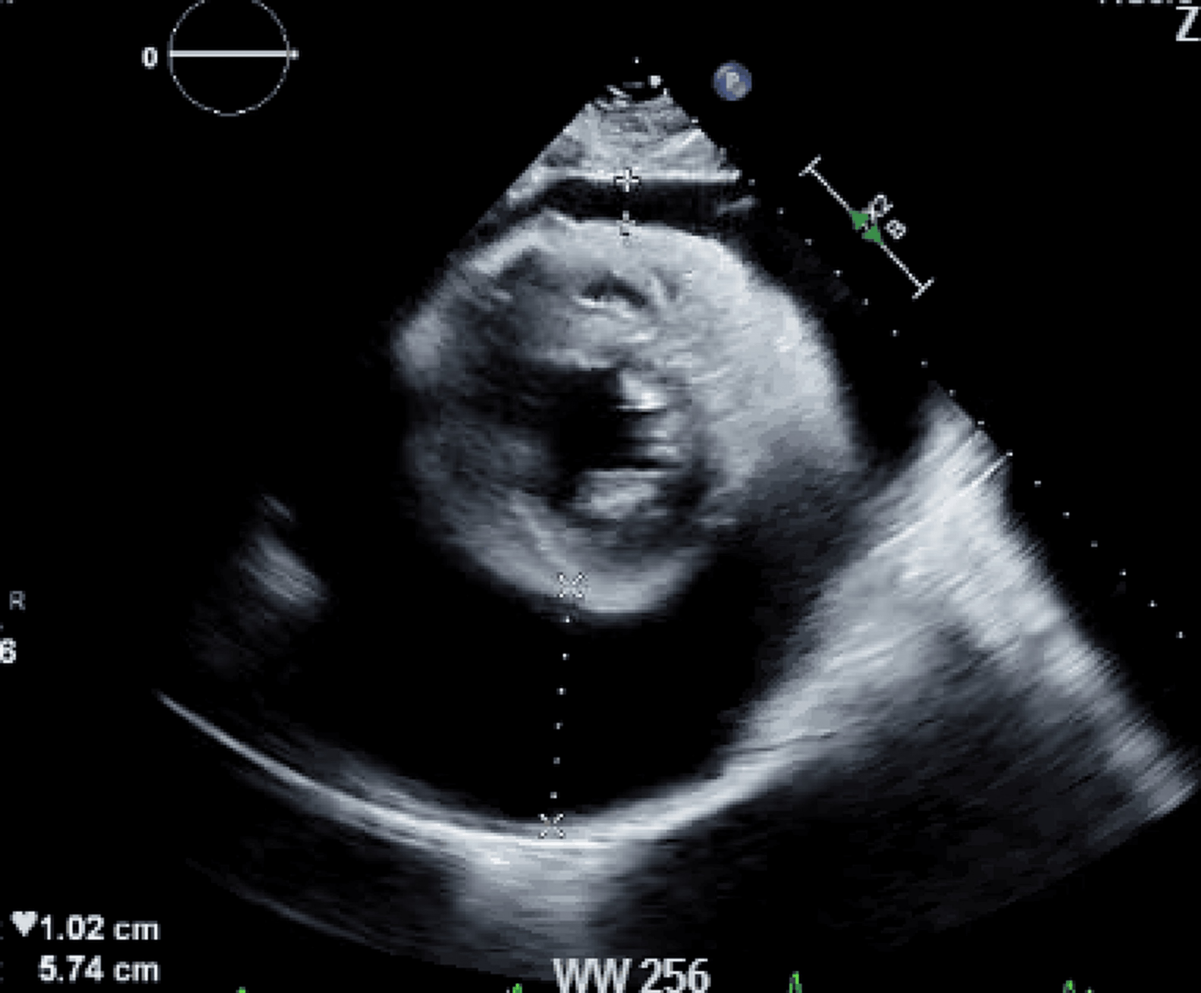
Transthoracic echocardiography showing circumferential anechoic space consistent with pericardial effusion.

**Figure 5:**
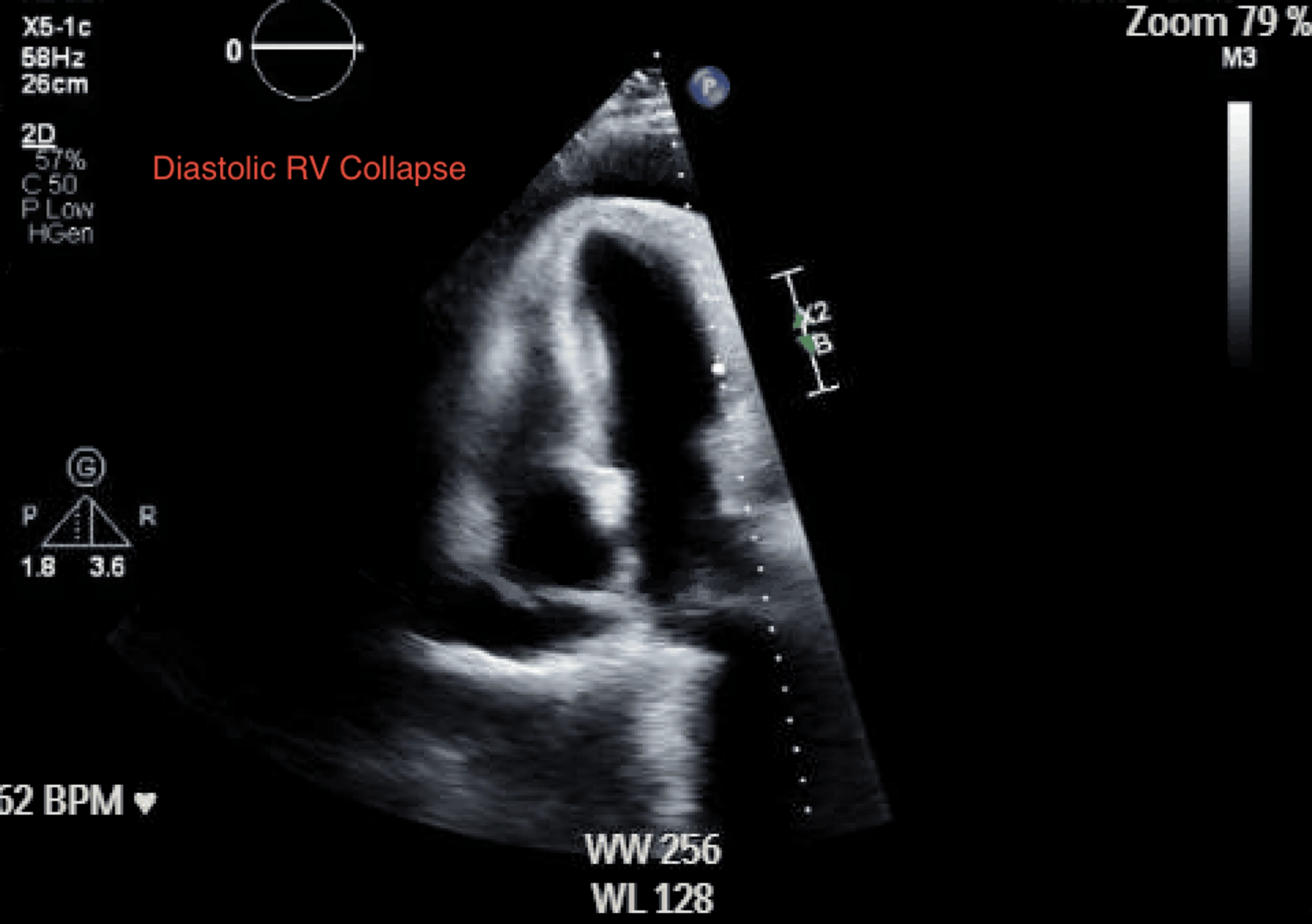
Transthoracic echocardiography showing early diastolic RV free-wall collapse consistent with tamponade physiology. RV: right ventricular

**Figure 6:**
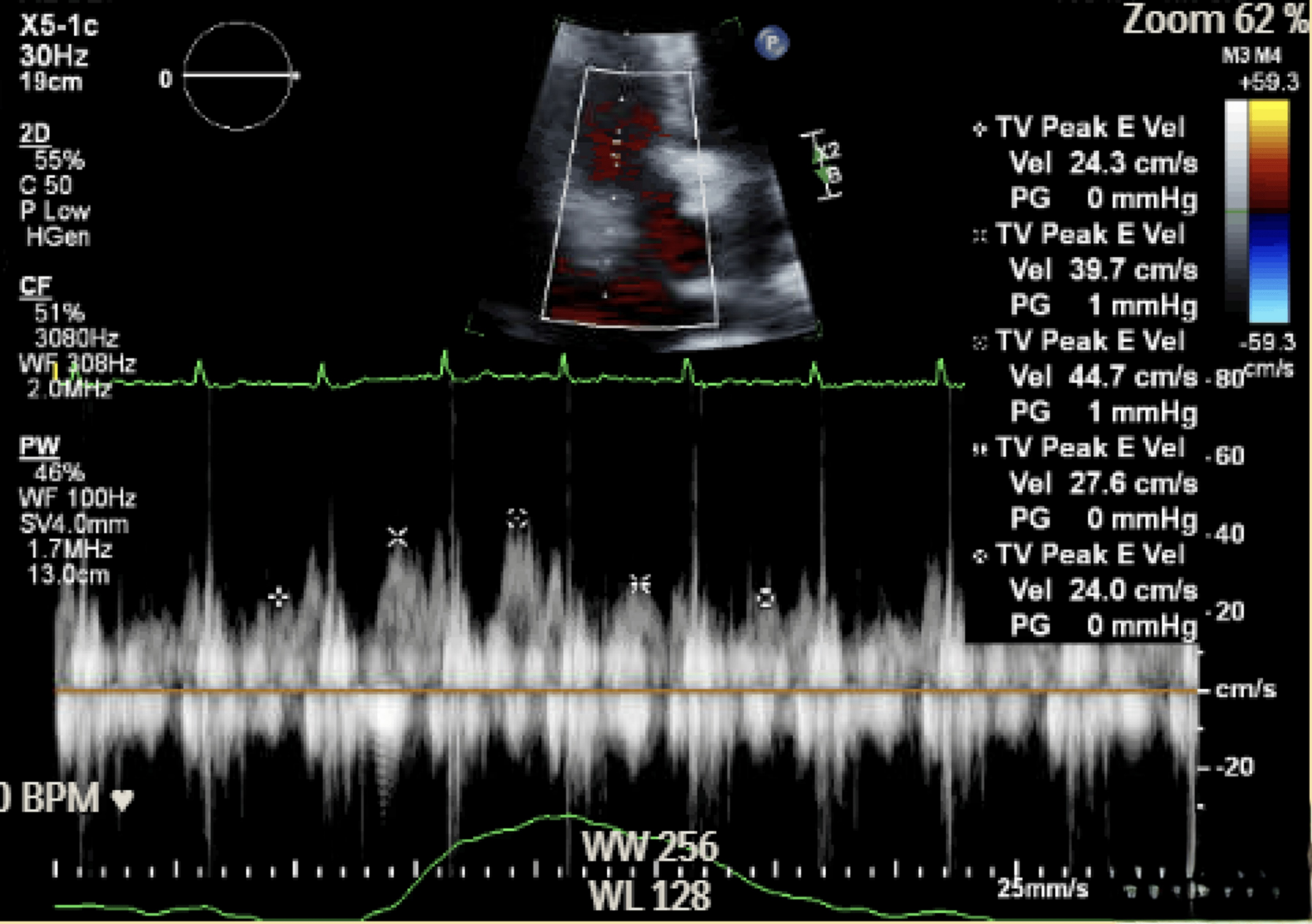
Doppler echocardiography showing marked tricuspid inflow respiratory variation supporting tamponade physiology.

Urgent transthoracic echocardiography revealed a large circumferential effusion with a swinging heart (Figure 4), right ventricular diastolic collapse (Figure 5), and marked respiratory variation of tricuspid inflow (Figure 6; approximately 46% respiratory variation in tricuspid E-wave peak velocity, 44.7 to 24 cm/s), with a plethoric inferior vena cava without respiratory variation, consistent with tamponade physiology. Endocrinology, cardiology, and thoracic surgery were consulted. The patient received intravenous fluids and stress-dose hydrocortisone 100 mg for empiric adrenal coverage during the initiation of intravenous levothyroxine, as thyroid hormone replacement can increase cortisol clearance and unmask occult adrenal insufficiency; glucocorticoids were discontinued after a reassuring morning cortisol (18.8 mcg/dL). She then underwent echo-guided emergent pericardiocentesis with the removal of three liters of straw-colored fluid; a pericardial catheter was left to gravity drainage and removed on hospital day 5 after declining output and imaging improvement.

Pericardial fluid analysis showed protein 5.4 g/dL, lactate dehydrogenase (LDH) 84 U/L, and glucose 75 mg/dL; cultures and cytology were negative, supporting a noninfectious, nonmalignant profile. Although Light's criteria are widely used for pleural effusions, they are not validated for pericardial fluid and may misclassify pericardial effusions; interpretation should rely on clinical context with cultures and cytology. Concurrent serum protein and LDH were not obtained; therefore, serum-to-fluid ratios could not be calculated. Screening serologies were limited but negative (antinuclear antibody and anti-Smith), as were hepatitis B and C antibodies; liver, renal, and adrenal function were normal, together favoring hypothyroidism as the etiology by exclusion. Key laboratory values and reference ranges are summarized in Table 1. Thyroid replacement was initiated with intravenous levothyroxine 200 μg daily for three doses, followed by oral levothyroxine 125 μg daily; intravenous therapy was used short-term to ensure reliable replacement in critical illness with hemodynamic instability, and the patient had no altered mental status or myxedema coma. Hemodynamics normalized, symptoms improved, and follow-up echocardiography showed only a minimal residual effusion without tamponade physiology.

**Table 1:** Key laboratory results with reference ranges. TSH: thyroid-stimulating hormone; LDH: lactate dehydrogenase; ANA: antinuclear antibody

Test	Result	Units	Reference range
TSH	65.04	μIU/mL	0.40-4.50
Free T4	<0.4	ng/dL	0.7-1.5
Morning cortisol	18.8	mcg/dL	10-20
Pericardial fluid protein	5.4	g/dL	No universal normal
Pericardial fluid LDH	84	U/L	No universal normal
Pericardial fluid glucose	75	mg/dL	No universal normal
ANA	Negative		Negative
Anti-Smith antibody	Negative		Negative
Hepatitis B antibody	Negative		Negative
Hepatitis C antibody	Negative		Negative

## Discussion

Pericardial effusion is a known manifestation of long-standing hypothyroidism, typically accumulating slowly and permitting very large volumes before pericardial reserve is exceeded [1,2]. While large effusions may develop in myxedema due to gradual accumulation, the three-liter volume drained in our patient is exceptional and highlights how prolonged untreated hypothyroidism can allow extreme pericardial fluid accumulation before clinical decompensation.

Severe hypothyroidism blunts sympathetic tone and β-adrenergic responsiveness, so the compensatory tachycardia seen in other tamponade etiologies may be attenuated, yielding bradycardic tamponade and a potential diagnostic pitfall [1,2,8]. Echocardiography remains central to diagnosis: large effusion with respiratory variation of atrioventricular (AV) valve inflow (≈≥25% mitral; ≥40% tricuspid) or right-sided chamber collapse with a plethoric inferior vena cava supports tamponade physiology [3,4]. In our patient, the combination of a large circumferential effusion and marked inflow variation prompted definitive drainage. Rapid evacuation of large pericardial effusions carries a recognized risk of pericardial decompression syndrome (PDS), a rare but potentially serious complication characterized by acute ventricular dysfunction and pulmonary edema following drainage, particularly in cases of massive, chronic effusions [5].

Assigning etiology requires a structured exclusion: infection and malignancy are addressed via cultures/cytology; biochemical panels (protein, LDH, glucose) help characterize the fluid while recognizing that conventional transudate/exudate cutoffs (Light's criteria) do not reliably apply to pericardial effusions [3]. Screening for autoimmune or viral causes should be tailored; our limited serologies (antinuclear antibody, anti-Smith, hepatitis B and C antibodies) were negative, and there were no clinical stigmata of connective tissue disease, making myxedema-related effusion most likely in the context of profound hypothyroidism [3]. Therapeutically, urgent pericardiocentesis with short-term catheter drainage is indicated when tamponade physiology is present, accompanied by careful endocrine management with adrenal coverage when appropriate and levothyroxine replacement (intravenous loading in critical illness followed by oral maintenance) [6-8]. These steps typically lead to rapid hemodynamic recovery and prevent recurrence as euthyroidism is restored.

## Conclusions

Severe, untreated hypothyroidism can cause massive pericardial effusion and, rarely, tamponade. In this setting, the expected reflex tachycardia of tamponade may be absent due to blunted sympathetic tone ("myxedema heart"), making reliance on bedside heart rate potentially misleading. Echocardiography is therefore pivotal to confirm tamponade physiology and guide urgent pericardial drainage, followed by thyroid hormone replacement to treat the underlying cause and reduce recurrence risk.
